# The Association of Proprotein Convertase Subtilisin/Kexin Type 9 to Plasma Low-Density Lipoproteins: An Evaluation of Different Methods

**DOI:** 10.3390/metabo11120861

**Published:** 2021-12-10

**Authors:** Laura Canclini, Amir Mohammad Malvandi, Patrizia Uboldi, Najoua Jabnati, Liliana Grigore, Alberto Zambon, Andrea Baragetti, Alberico Luigi Catapano

**Affiliations:** 1 Department of Pharmacological and Biomolecular Sciences, University of Milan, 20133 Milan, Italy; laura.canclini@unimi.it (L.C.); patrizia.uboldi@unimi.it (P.U.); najoua.jabnati@studenti.unimi.it (N.J.); andrea.baragetti@unimi.it (A.B.); 2 IRCCS Multimedica, 20138 Milan, Italy; amir.malvandi@multimedica.it; 3 IRCCS Multimedica, 20099 Sesto San Giovanni, Italy; grigore.centroatero@gmail.com (L.G.); alberto.zambon@unipd.it (A.Z.); 4 Center for the Study of Atherosclerosis, Bassini Hospital, 20092 Cinisello Balsamo, Italy

**Keywords:** PCSK9, lipids, LDL, cardiovascular diseases, atherosclerosis, lipoproteins

## Abstract

Proprotein convertase subtilisin/kexin type-9 (PCSK9) is key regulator of low-density lipoprotein (LDL) metabolism. A significant proportion of PCSK9 is believed to be associated with LDL in plasma as it circulates, although this finding is still a matter of debate. The purpose of this study was to establish an experimental method to investigate the presence of such an interaction in the bloodstream. We compared a number of well-established methods for lipoprotein (LP) isolation to clarify whether PCSK9 associates differently to circulating lipoproteins, such as KBr gradient ultracentrifugation, physical precipitation of ApoB-LPs, fast protein liquid chromatography (FPLC) and iodixanol gradient ultracentrifugation. Our data show heterogeneity in PCSK9 association to lipoproteins according to the method used. Two methods, iodixanol ultracentrifugation and column chromatography, which did not involve precipitation or high salt concentration, consistently showed an interaction of PCSK9 with a subfraction of LDL that appeared to be more buoyant and have a lower size than average LDL. The percent of PCSK9 association ranged from 2 to 30% and did not appear to correlate to plasma or LDL cholesterol levels. The association of PCSK9 to LDL appeared to be sensitive to high salt concentrations. FPLC and iodixanol gradient ultracentrifugation appeared to be the most suitable methods for the study of this association.

## 1. Introduction

The causal role of low-density lipoproteins (LDL) in cardiovascular diseases (CVD) is well known [1]. Lowering plasma LDL-cholesterol (LDL-C) translates to a proportional reduction in cardiovascular risk [2]. LDL clearance from the circulation primarily occurs in the liver through the LDL receptor (LDLR) pathway [3]. PCSK9 promotes intracellular LDLR degradation, leading to fewer LDLRs on the cell membrane and decreased LDL uptake [4]. PCSK9 inhibition with monoclonal antibodies lowers LDL-C levels by ∼60%, resulting in a substantial reduction in cardiovascular risk [5]. Several lines of evidence suggest a possible PCSK9–LDL association [6]. Indeed, more than half of the PCSK9 is removed along with LDL by LDL-apheresis treatment, suggesting a possible PCSK9–LDL interaction [7]. Size-exclusion chromatography of human plasma confirmed this as it showed that PCSK9 co-eluted with LDL-sized particles [8], and up to 40% of the total PCSK9 was recovered in the LDL fraction derived from density gradient centrifugation [6]. Conversely, LDLs isolated with the routine KBr ultracentrifugation were free from PCSK9 [9]. A possible PCSK9 association with Lp(a) has also been suggested [10], however fast protein liquid chromatography (FPLC) showed PCSK9 plasma distribution to be inconsistent with Lp(a) interaction [11]. More controversial is the association of PCSK9 to high-density lipoproteins (HDLs). The fact that PCSK9 lipoprotein distribution is maintained in subjects carrying genetic HDL defects suggests that HDL does not contribute to PCSK9 transport in plasma [11]; however, the incubation of hPCSK9 with mouse serum showed that PCSK9 associated with HDL [12].

In this work we aimed to establish an experimental procedure to study the possible PCSK9 association, in vivo, with plasma LDL. To this aim, we quantified PCSK9 association with LDL with different biochemical approaches to identify the best condition to be used for further biological and clinical studies. We showed that PCSK9 association with LDL existed and is sensitive to high salt concentration. Both FPLC and iodixanol gradient ultracentrifugation were suitable methods for the study of PCSK9’s molecular associations.

## 2. Results

### 2.1. KBr-Based Methods

**Figure 1:**
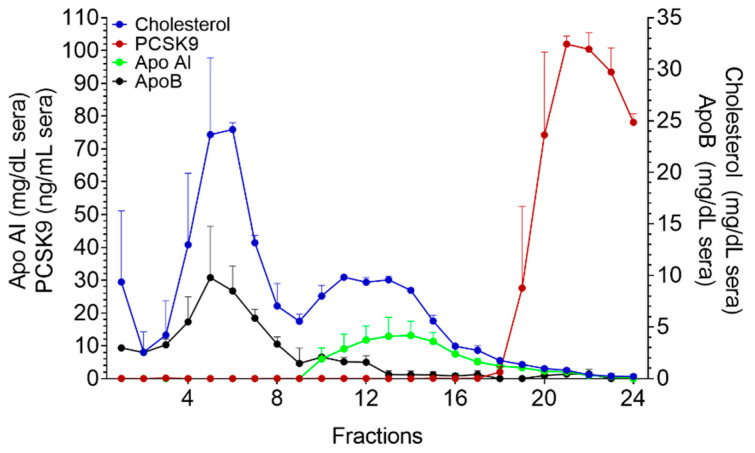
**KBr gradient ultracentrifugation.** Cholesterol, PCSK9, ApoB and Apo AI profile of LP fractions isolated with KBr gradient (n = 4). Data were reported as mean + SEM.

KBr sequential ultracentrifugation is commonly referred as the gold standard method for LP separation [13]. In line with previous reports [9,14], our evaluation showed no detectable PCSK9 in the lipoprotein fractions obtained by this method (data not shown). To answer whether the stepwise separation induced loss of PCSK9, we switched to a one-step KBr gradient ultracentrifugation (UC) method. LPs were isolated from serum according to a previously described protocol [15] with some minor modifications. Our results showed that negligible amounts of PCSK9 were traceable in LP fractions (Figure 1).

### 2.2. Precipitation of ApoB-LPs with Phosphotungstic Acid and MgCl_2_

**Figure 2:**
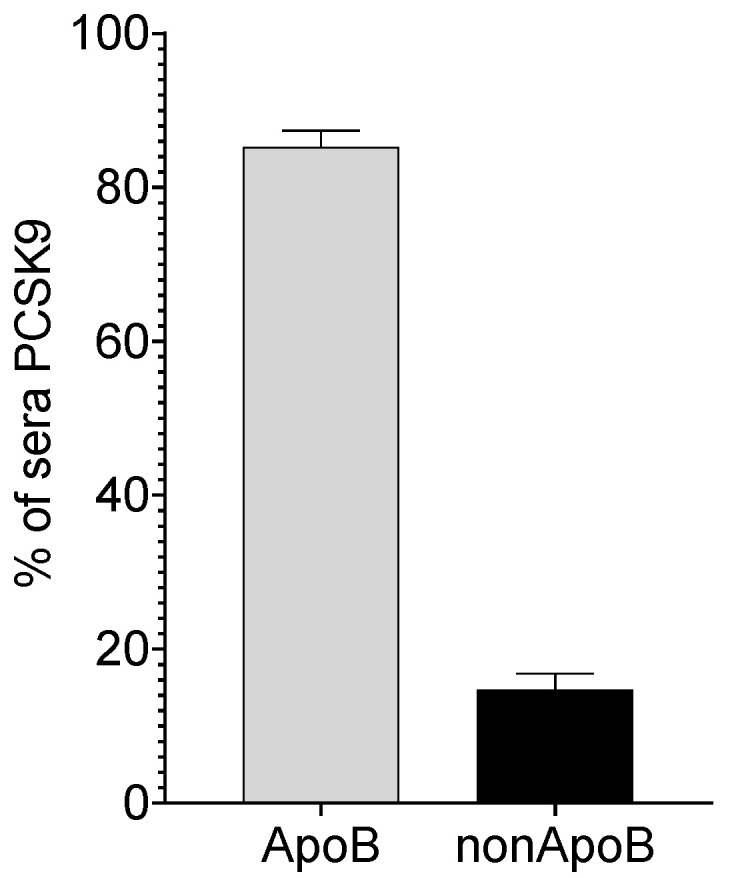
**Physical precipitation of ApoB-LPs.** PCSK9 was quantified in the supernatant. The PCSK9 content of the ApoB lipoproteins was derived by difference from total plasma PCSK9 values (n = 4). Data were reported as mean + SEM.

Several procedures for LP isolation from human serum by physical precipitation with polyanions and divalent cations are described [16]. With a precipitating solution made of phosphotungstic acid and MgCl_2_, we obtained a precipitate made of the ApoB-containing LPs and a supernatant, corresponding to the non-ApoB fraction of sera. We recovered around 80% of the PCSK9 into the ApoB precipitate (Figure 2). This finding was clearly at odds with all previous reports with other methods and probably reflected unspecific interactions due to the precipitant, which might have either interfered with the assay or non-specifically precipitated PCSK9. Preliminary data suggested that the precipitation and lipoprotein aggregation per se contributed to the precipitation of free PCSK9 (not shown).

### 2.3. Fast Protein Liquid Chromatography

**Figure 3:**
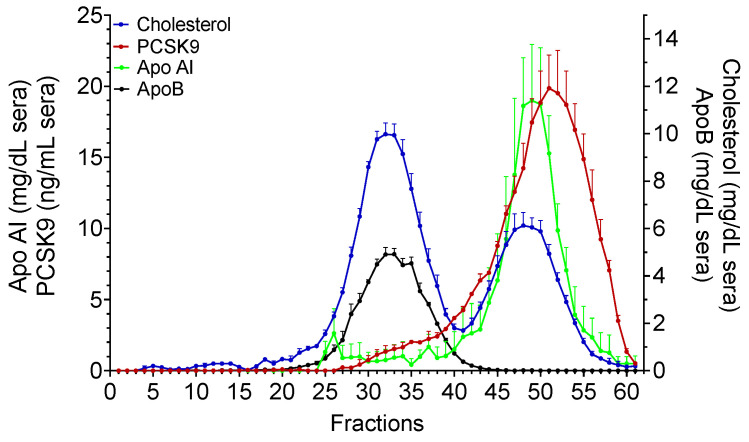
**FPLC.** Cholesterol, ApoB, Apo AI (n = 10) and PCSK9 (n = 7) profiles of FPLC fractions. Data were reported as mean + SEM.

To determine the association of PCSK9 with LDL under conditions avoiding high salt concentrations, we performed size-exclusion chromatography (FPLC) fractionation. PCSK9 protein levels in the FPLC fractions were quantified by ELISA, as described in the Materials and Methods section. PCSK9 showed a main peak (fractions 45–60) (Figure 3), indicating that the majority of PCSK9 circulated as free forms or that it was associated with HDL or other components of the serum. A small portion of PCSK9 (approx. 5–10% of total recovered PCSK9) eluted within the LDL size range (fractions 29–40), which suggested a PCSK9–LDL association. This percentage was in line with previous reports [8,11].

### 2.4. Iodixanol Gradient Ultracentrifugation (OptiPrep)

**Figure 4:**
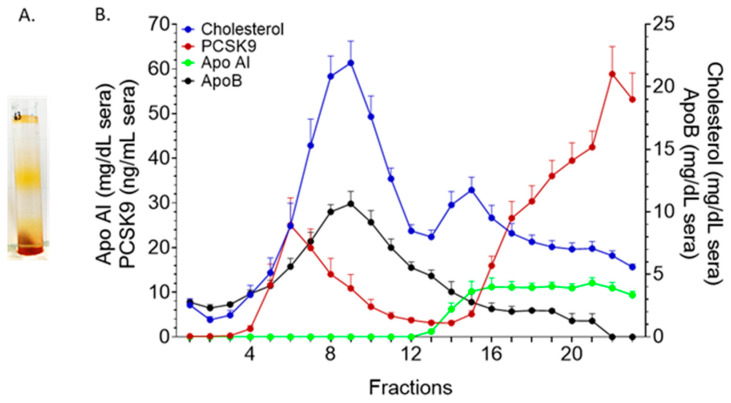
**Iodixanol gradient Ultracentrifugation.** (**A**) LP separation in OptiPrep gradient, (**B**) cholesterol, PCSK9, ApoB and Apo AI profile of OptiPrep fractions (n = 6). Data were reported as mean + SEM.

We used floatation ultracentrifugation in iodixanol gradient to isolate the LP fractions and assess their association with PCSK9. We modified a previously described protocol [6] to improve LDL separation: with a 4-layered gradient we resolved serum into a light fraction (containing VLDL), followed by a medium-density fraction (corresponding to LDL) and a heavy-density fraction (that contained HDL and other components of the serum) (Figure 4A). Our results showed a peak in PCSK9 in the less dense LDL sub-fractions (Figure 4B, fractions 4–9). The percentage of association was around 22.4 ± 8.3% (mean ± SD) of the total PCSK9 recovered. A large portion of PCSK9 was in the fractions with higher density while it was not detected in the VLDL containing fractions.

### 2.5. PCSK9 Associates with ApoB-LPs with Its Active Form

**Figure 5:**
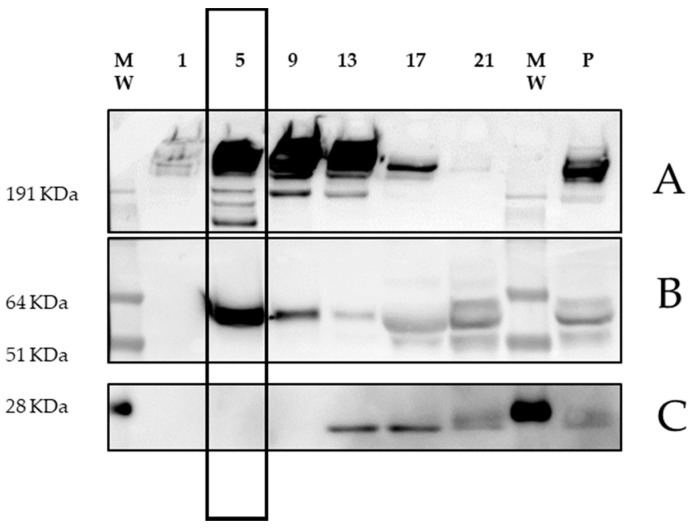
**Analysis of OptiPrep fractions.** Representative immunoblotting of ApoB (**panel A**), PCSK9 (**panel B**) and Apo AI (**panel C**) of the pooled OptiPrep fractions. MW: Prestained protein standard; 1: POOL 1-3; 5: POOL 5-8; 9: POOL 912; 13: POOL 13-16; 17: POOL 17-20; 21: POOL 21--24; P: plasma. PCSK9 peak in LDL (fractions 5–8) corresponds to the highlighted band.

To further characterize the PCSK9–LDL association, we analyzed, by immunoblotting, the pooled OpitPrep fractions as described in the Material and Methods section. In plasma (Figure 5—“P”) and in the heaviest OptiPrep fractions we detected a PCSK9 precursor (≈74 KDa), PCSK9 in its mature form (≈62 KDa) and in its truncated form (≈55 KDa) (Figure 5, panel B). Our results confirmed PCSK9 association with the lightest LDL sub-fractions and showed that PCSK9 associated with LDL through its active form (Figure 5, panel B—“fractions 5–8”). These observations were in line with previously reported data [7]. We did not find PCSK9 in the first fractions (Figure 5, panel B—“1”) containing VLDL. The PCSK9 found at higher densities clearly showed the presence of both the intact and cleaved forms (see fraction 17 and 21).

## 3. Discussion

In this study, we investigated the association of PCSK9 with LDL based upon previous discordant data regarding their possible interaction. Robust procedures for LP isolation are essential to gain a better insight into their interactions and physiological modulation. Herein, we compared different methods for LP isolation to be used for the study of their possible association with PCSK9.

Our results indicated that high salt concentrations could disrupt the interaction of PCSK9 with lipoproteins. This was reported previously for some components of plasma LPs, which were dissociated upon increasing salt concentrations [17], and the literature reports showed no PCSK9 association with LDL upon density gradient ultracentrifugation with high salt concentrations [9,14]. We believe that the physical precipitation of the ApoB-LP was not a valid method for studying the PCSK9 interactions due to the precipitant, which may have either interfered with the assay or non-specifically precipitated PCSK9. Additionally, this method did not allow separation of the different ApoB-LP subtypes, thus hampering a clear understanding of the association of PCSK9 to lipoprotein subfractions.

Size-exclusion chromatography of the serum provided an LP separation at high resolution, without interfering with their interactions. Our results showed PCSK9 co-eluted with LDL.

Isolation of the LPs according to their different densities in a neutral density gradient (OptiPrep ultracentrifugation) resulted in a comparable distribution pattern, with 22.4 ± 8.3% of the total recovered PCSK9 found in the LDL fractions. These data suggested the presence of a PCSK9–LDL association. The % of association varied from 2% to 30% of the total recovered PCSK9.

Interestingly, lipoprotein separation with FPLC resulted in a peak of PCSK9 that was not superimposable with the main LDL peak (Figure 3, fractions 30–40). The same finding was present after OptiPrep separation (Figure 4, fractions 4–9 and 4–12, respectively). Altogether, these data suggested that PCSK9 circulated in association with a specific LDL sub-fraction in its active, mature form (Figure 5). This latter finding posed the question as to whether the subfraction of LDL specifically binding to PCSK9 possessed specific structural and biological characteristics that could inform on the area of PCSK9 that is required to interact with LDL. Our data from SDS gel electrophoresis suggested a different apolipoprotein pattern for this subfraction, and also that the LDL fraction that interacted with PCSK9 was enriched with triglycerides (not shown).

## 4. Conclusions

In summary, we proposed FPLC and iodixanol based ultracentrifugation as two methods for the study of PCSK9 association with LDL. Further we have shown that a specific subset of LDL binds to PCSK9 in its mature form. Whether PCSK9 interacted with a specific subcomponent, which was present only in a fraction of isolated LDL, bears a physiological significance that remains to be addressed. LDL interaction with PCSK9 is, in fact, believed to modulate the activity of PCSK9 towards the LDLR [18], and we are currently investigating the possible biological function of this LDL subfraction as well the detailed lipidomic and proteomic. Discovering the regulatory mechanisms that affect PCSK9 activity represents a possibility for the development of new lipid-lowering therapies. Determining the functional outcome of the PCSK9–LDL association could shed some light on such mechanisms. Our results provided the basis for a better understanding of the physiology of PCSK9 and the mechanisms underlying hypercholesterolemia.

## 5. Materials and Methods

### 5.1. Subjects and Samples

We recruited fasting volunteers. Table 1 summarizes their baseline characteristics. All the participants were informed and signed the consensus letter. Participation in the study was voluntary and anonymous, according to the approval of the institutional ethical committee.

Blood samples were collected using sterile venipuncture tubes. Serum and plasma were obtained by low-speed centrifugation and immediately supplemented with protease inhibitor (Halt^TM^ Protease Inhibitor Cocktail, Thermo Fisher Scientific, Waltham, MA, USA). Cholesterol was measured using the clinical grade reagent (HORIBA Scientific Ltd., Kyoto, Japan) with GloMax^®^ Discover Microplate Reader (Promega, Madison, WI, USA). ApoB and Apo AI were quantified with immunoturbidimetry assays, for KBr and OptiPrep fractions (LP3839 for ApoB and LP3838 for Apo AI from Randox laboratories Ltd., Crumlin, UK) or with ELISA (Mabtech, Nacka Strand, Sweden), for FPLC fractions. PCSK9 was quantified with the commercially available Quantikine Human Proprotein Convertase 9/PCSK9 ELISA Kit (R&D systems, Minneapolis, MN, USA), according to the manufacturer instructions. In the case of need, collected fractions were kept at −80 °C.

### 5.2. KBr Gradient Ultracentrifugation

The density of serum was adjusted to 1.25 with solid KBr (0.398 g/mL serum). A volume of 3 mL of the density adjusted serum was added to the centrifuge tube (Ultra-clear certified particle-free, Beckman-Coulter, Brea, CA, USA), followed by careful stratification of 2 mL 1.225 g/mL KBr solution, 4 mL 1.100 g/mL KBr solution and 3 mL of 1.006 g/mL of KBr solution. The tubes were centrifuged for 18 h at 40,000× *g* rpm at 4 °C in an SW 41 Ti rotor (Beckman Coulter, Brea, CA, USA) in a Beckman Coulter ultracentrifuge Optima L-100K (Beckman Coulter, Brea, CA, USA). Their content was manually divided into 0.5 mL fractions, which then were quantified for their ApoB, Apo AI, PCSK9 and cholesterol content.

### 5.3. Phosphotungstic Acid Precipitation of ApoB-LPs

100 µL of the precipitating solution (phosphotungstic acid (40 g/L) and MgCl2 (100 g/L) brought at pH 6.2) was added to 1 mL of fresh serum, followed by 30 s of vortexing and 10 min of rest at room temperature. The precipitated ApoB fraction was obtained by centrifugation for 15 min at 1500× *g*. We quantified the PCSK9 content of the supernatant (non-ApoB) with ELISA; the value of the Apo-B part was obtained by calculating the difference from the serum value.

### 5.4. Fast Protein Liquid Chromatography (FPLC)

We used a Superose 6 column (GE Healthcare, Chicago, IL, USA) on an NGC^TM^ chromatography system FPLC (BioRad laboratories Inc., Hercules, CA, USA) with an eluent solution consisting of 0.15 M NaCl pH 7.2 + 0.01% EDTA + 0.02% Sodium azide. A volume of 0.5 mL of sera was filtered (0.45 microns; Merk Millipore, Burlington, MA, USA) and loaded on the device. The system was kept at a constant flow rate of 0.75 mL/min. We collected 0.75 mL fractions, which we analyzed for their cholesterol and PCSK9 content. Analysis of ApoB and Apo AI on the fractions were done using ELISA (Mabtech, Nacka Strand, Sweden).

### 5.5. Iodixanol Density Gradient Ultracentrifugation (OptiPrep)

Lipoproteins were obtained using a four-layered OptipPrep^TM^ density gradient (60% *w*/*v* iodixanol, Stemcell Technologies, Vancouver, BC, Canada) in Ultraclear centrifuge tubes (Beckman Coulter, Brea, CA, USA). The composition of the gradient is summarized in Table 2. Briefly, the denser plasma-OptiPrep^TM^ solution (14%) was layered below the 10% iodixanol solution using a sterile syringe needle (18 gauge × 6 mm, Sigma-Aldrich, St. Louis, MO, USA). An 8% iodixanol density was subsequently stratified on the denser layer. Furthermore, the tube was filled up with 1.5 mL of Tris (Trizma base, Sigma-Aldrich, St. Louis, MO, USA) solution (10 mM, pH 7.4). The complete gradient was centrifuged with an SW 41 Ti rotor spun at 40,000× *g* rpm for 18 h at 4 °C, in a Beckman Coulter ultracentrifuge Optima L-100K (Beckman Coulter, Brea, MA, USA). Measured 0.5 mL fractions were manually collected, starting from the upper layer.

### 5.6. Immunoblot of OptiPrep Fractions

OptiPrep fractions were pooled and prepared in LDS sample buffer (Thermo Fisher Scientific, Waltham, MA, USA) under reducing conditions (2-Mercaptoethanol 2.5%). Samples were separated by electrophoresis using NuPAGE^TM^ Mini Protein Gel (4 to 12%, Bis-Tris, 1.5 mm, Thermo Fisher Scientific (Invitrogen) Waltham, MA, USA). ApoB and PCSK9 gels were transferred to nitrocellulose membrane (0.45 µm, BioRad laboratories Inc., Hercules, CA, USA); Apo AI gel was transferred to PVDF membrane (0.45 µm, Amersham^TM^, GE Healthcare Life Sciences Chicago, IL, USA). Human ApoB was detected with a mouse anti-human ApoB antibody (MABTECH, 3715-3-250); PCSK9 was detected with a rabbit anti-human PCSK9 antibody (ABGENT, AP20995c); Apo AI was detected with a mouse anti-human Apo AI antibody MABTECH, (3710-3-250). Secondary antibodies HRP conjugated anti-mouse (NA931-1ML) and anti-rabbit (NA934-1ML) were purchased from GE Healthcare (Amersham^TM^, GE Healthcare Life Sciences, Chicago, IL, USA). The signal was detected with an ultra-sensitive enhanced chemiluminescent substrate (Thermo Fisher Scientific, Waltham, MA, USA) and acquired with UVTEC (UVITEC, Cambridge, UK).

## Figures and Tables

**Table 1:** Baseline characteristics of the subjects enrolled. Data were reported as mean ± SEM (standard error of the mean).

Parameter	Men (n = 6)	Women (n = 13)
Total cholesterol (mg/dL)	182 ± 15	168 ± 5
Triglycerides (mg/dL)	96 ± 29	74 ± 7
LDL-cholesterol (mg/dL)	105 ± 11	103 ± 5
HDL-cholesterol (mg/dL)	58 ± 2	56 ± 3
PCSK9 (ng/mL)	380 ± 40	300 ± 13

**Table 2:** OptiPrep densities.

Material	14% OptiPrep-Serum Density	10% OptiPrep Density	8% OptiPrep Density
OptiPrep^TM^	1.28 mL	0.42 mL	0.33 mL
Trizma base 10 mM pH 7.4	1.42 mL	2.08 mL	2.17 mL
Serum	2.8 mL	-	-
Total Volume	5.5 mL	2.5 mL	2.5 mL

## Data Availability

Data are available upon request from ALC.
